# Footprints of Directional Selection in Wild Atlantic Salmon Populations: Evidence for Parasite-Driven Evolution?

**DOI:** 10.1371/journal.pone.0091672

**Published:** 2014-03-26

**Authors:** Ksenia J. Zueva, Jaakko Lumme, Alexey E. Veselov, Matthew P. Kent, Sigbjørn Lien, Craig R. Primmer

**Affiliations:** 1 Department of Biology, University of Turku, Turku, Finland; 2 Department of Biology, University of Oulu, Oulu, Finland; 3 Institute of Biology, Karelian Research Centre of RAS, Petrozavodsk, Russia; 4 Centre for Integrative Genetics (CIGENE) and Department of Animal and Aquacultural Sciences, Norwegian University of Life Sciences, Ås, Norway; Swansea University, United Kingdom

## Abstract

Mechanisms of host-parasite co-adaptation have long been of interest in evolutionary biology; however, determining the genetic basis of parasite resistance has been challenging. Current advances in genome technologies provide new opportunities for obtaining a genome-scale view of the action of parasite-driven natural selection in wild populations and thus facilitate the search for specific genomic regions underlying inter-population differences in pathogen response. European populations of Atlantic salmon (*Salmo salar L.*) exhibit natural variance in susceptibility levels to the ectoparasite *Gyrodactylus salaris* Malmberg 1957, ranging from resistance to extreme susceptibility, and are therefore a good model for studying the evolution of virulence and resistance. However, distinguishing the molecular signatures of genetic drift and environment-associated selection in small populations such as land-locked Atlantic salmon populations presents a challenge, specifically in the search for pathogen-driven selection. We used a novel genome-scan analysis approach that enabled us to i) identify signals of selection in salmon populations affected by varying levels of genetic drift and ii) separate potentially selected loci into the categories of pathogen (*G. salaris*)-driven selection and selection acting upon other environmental characteristics. A total of 4631 single nucleotide polymorphisms (SNPs) were screened in Atlantic salmon from 12 different northern European populations. We identified three genomic regions potentially affected by parasite-driven selection, as well as three regions presumably affected by salinity-driven directional selection. Functional annotation of candidate SNPs is consistent with the role of the detected genomic regions in immune defence and, implicitly, in osmoregulation. These results provide new insights into the genetic basis of pathogen susceptibility in Atlantic salmon and will enable future searches for the specific genes involved.

## Introduction

Parasites are considered to be among the strongest selective forces driving the evolution of host populations (reviewed by [1], [2]). The efficiency of the host response following exposure to a novel pathogen can vary considerably [3], [4] and can even result in local extinction [5]. Moreover, the formation of host defence may follow different evolutionary strategies and take the form of either resistance (decreases parasite fitness) or tolerance (increases the ability to cope with parasite-induced diseases), and thus, different strategies of host defense can have different effects on parasite virulence [6], [7]. Given that novel pathogens tend to increasingly emerge in natural populations due to environmental changes [8] and human-related activities [9], understanding the mechanisms underlying the formation of host adaptation is of utmost importance. Nevertheless, revealing the genetic basis of the evolution of pathogen resistance or tolerance is still challenging. During recent decades, the major histocompatibility complex (MHC) genes have been a target of studies focused on investigating how natural selection can facilitate adaptive immune response at the gene level in vertebrates (rev. by [10], [11]). However, a much broader range of immune-relevant loci may also be important [12], [13]. Recent developments in genomic technologies have enabled genome-wide scale approaches for the identification of genes and gene networks affected by natural selection [14]–[16]. These approaches have been also used in the immunological research of wild species, including salmon microarray studies (see below).

Due to their commercial importance and their increasing significance in aquaculture, Atlantic salmon have been the target of considerable research, including a considerable focus on the molecular basis of aquaculture strain response to various diseases that have emerged in hatchery facilities (e.g. [17]). This research includes studies on the genetic basis of resistance to infectious pancreatic necrosis [18]–[20], anaemia virus [21], [22] and furunculosis-causing bacteria *Aeromonas salmonicida* [17], [23], [24], with a focus on MHC diversity. Another line of research has focused on the molecular response to acute pathogen exposure at the RNA level using Atlantic salmon cDNA microarray data [25]–[27]. However, studies of the evolutionary responses of wild populations to novel pathogen exposure at a genome-wide scale are sorely lacking, not just in Atlantic salmon but in wild species in general.

One of the most remarkable examples of the evolution of tolerance and/or resistance to a potentially harmful parasite has been observed in Atlantic salmon (*Salmo salar*), with populations differing widely in their susceptibility to the monogenean ectoparasite *Gyrodactylus salaris*. Stocking of salmon originating from one basin to another has inadvertently exposed numerous populations of Atlantic salmon in Norway and one population in Russia to this ectoparasite and subsequently decimated them (rev. by [28]). Subsequent investigations have indicated that Atlantic Ocean basin populations, including Barents Sea, White Sea and Norwegian coast populations, are susceptible to *G. salaris*, with mortality rates of up to 95% being commonly reported [29]. In contrast, salmon from the Baltic Sea basin naturally coexist with the parasite and show moderate resistance, with approximately 20% of fish being infected [30] but having little or no negative effect on the host [31]. Further, landlocked populations from large lakes in northwest Russia are almost completely resistant, with low-level infections being observed in just 1% of fish [30].

The tolerance and near-resistance of Baltic and landlocked populations is thought to have evolved as a result of the different recolonization routes in northern Europe after the last glacial maximum. During the retreat of the Scandinavian Ice Sheet from its last Weichselian maximum (17,000-15,000 years ago), large freshwater reservoirs began to form [32]. Lake Onega was formed among the first, approximately 13,000 ya, followed by the Baltic Ice Lake, the predecessor of the modern Baltic Sea and Lake Ladoga [32], [33]. The resistant Onega and Ladoga lake populations were colonised by salmon that have been coevolving with the parasite in the eastern freshwater refugium for up to 132,000 years [30]. Modern tolerant Baltic salmon mostly descended from the same refugium, but possibly was supplemented with gene flow from Atlantic Ocean populations when the Baltic Sea gained its current form. Finally, highly susceptible salmon from the Barents and White Seas were not exposed to *G. salaris* until very recently [34]–[36]. One exception to this general scenario appears to be a single land-locked population of Pistojoki river (lake V. Kuito), which exhibits *G. salaris* resistance and is thought to be most closely related to the White Sea Atlantic salmon lineage [37].

The gradient in *G. salaris* resistance observed in north European Atlantic salmon offers an opportunity to use an evolutionary approach to study the genetic basis of parasite resistance. This, combined with the availability of an Atlantic salmon SNP chip [38], allows application of a genome scan approach to detect signatures of *G. salaris-*induced selection in the Atlantic salmon genome via approaches such as hitchhiking mapping [39]. Under a scenario of strong directional selection, which we assume to be the case in pathogen-threatened populations, the frequency of advantageous alleles and linked sites increases along with a simultaneous reduction in variability, a process known as a selective sweep (rev. by [40]). If the target of the selection pressure is different in the studied populations, then along with a reduction of variability within the population, genetic divergence between populations would increase: loci subject to directional selection would show larger differences between populations compared to non-selected loci [41]. Hitchhiking has proved to be a powerful method for identifying or strengthening candidate genomic regions in a broad range of species (e.g., zebrafish [42], threespine sticklebacks [43], dogs [44], human [45]). Importantly, the described approaches are suitable for detecting selection that has occurred up to 3,200 generations ago [46], which for Atlantic salmon would be approximately 13,000 years before present: precisely the timing of post-glacial selection.

There are, however, several challenges for detecting *G. salaris-*induced selection in Atlantic salmon populations. First, earlier research has indicated that Russian landlocked salmon populations, i.e., those with the highest level of *G. salaris* resistance, have relatively low population sizes and are hence strongly affected by genetic drift [36], [47]. Genetic drift can leave genomic “footprints” similar to natural selection, i.e., increased divergence and decreased diversity, albeit at a genome-wide scale. Thus, identification of signals of selection is expected to be more challenging in populations with a small effective population size [44], [48]. Even though it is challenging to distinguish between genomic signals of drift and selection, a promising approach is to compare replicate events in search for a parallel signal: in contrast to directional selection, one would not expect drift to cause the same patterns multiple times in independent comparisons.

A second challenge for detecting *G. salaris-*induced selection in north European salmon populations results from the fact that the *G. salaris* resistance gradient co-varies with several environmental gradients, one of the most prominent being salinity [13]. Therefore, distinguishing between the selective effects of *G. salaris* exposure and, e.g., salinity tolerance may be difficult. However, the phylogeographic history of the populations in the region enables the design of specific comparisons to potentially separate the signals of these alternative selective forces.

In this study, we used a novel genome-scan analysis approach aimed at i) identifying signals of selection in salmon populations affected by varying levels of genetic drift and ii) separating the potentially selected loci into those affected by pathogen (*G. salaris*)-driven selection and selection acting upon other environmental characteristics.

## Materials and Methods

### Ethics statement

Samples used in this study were obtained according to relevant national legislations and have been described previously [37].

### Sampled populations

**Figure 1:**
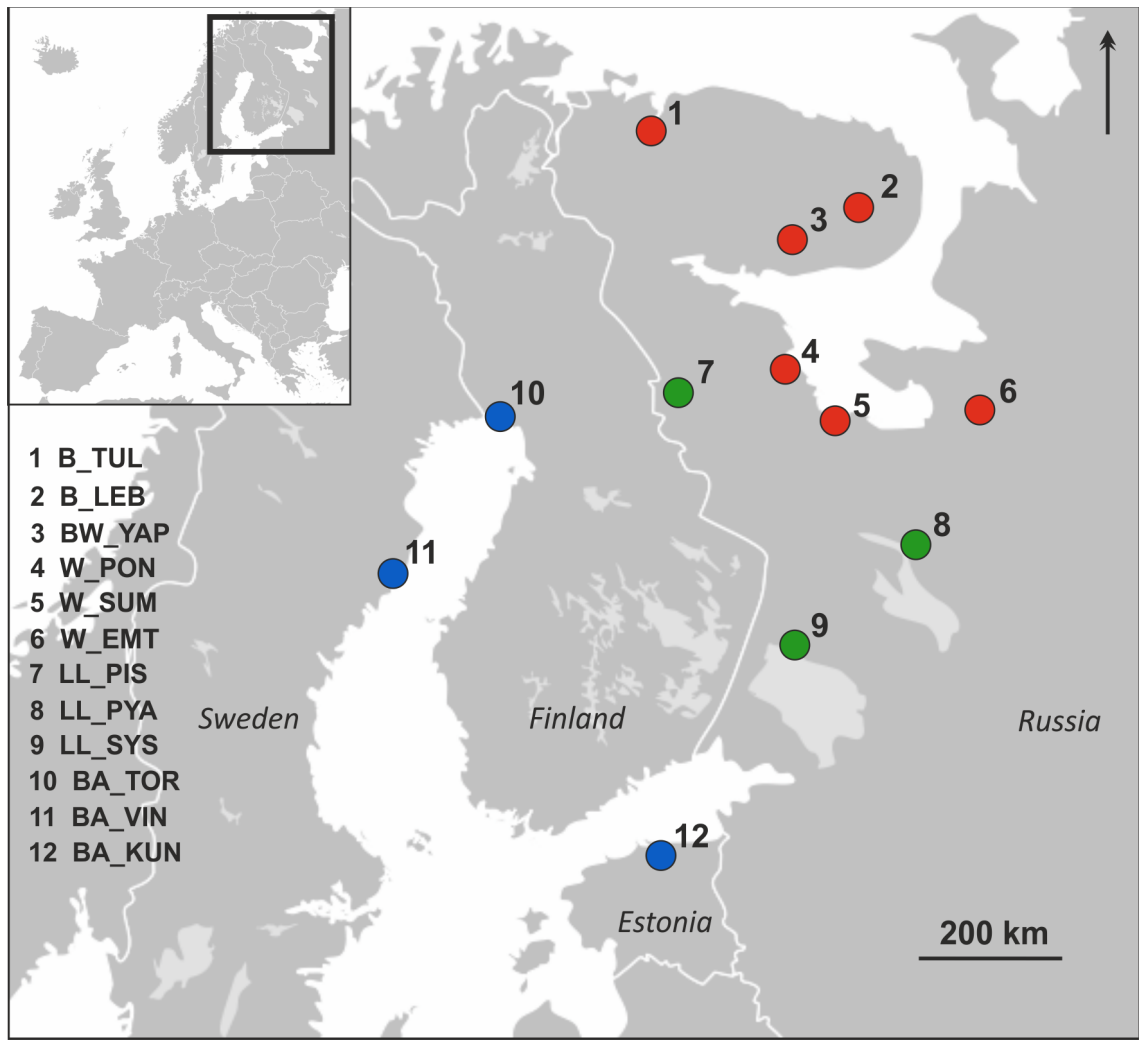
Map of northern Europe indicating the study populations: anadromous Atlantic Ocean, *G. salaris* susceptible (red); anadromous Baltic, moderately resistant (blue); landlocked, resistant to the parasite (green).

A total of 472 Atlantic salmon individuals representing 12 north European populations (Table 1, Figure 1) were included in the analysis. Population samples generally consisted of one to three year old parr, electrofished from a single river stretch of 100 –150 m^2^ at one time point between the years 1997 and 2005. Fin tissue was stored in 96.5% ethanol for subsequent DNA extraction. The sampled populations belong to different basins: the Barents and White Seas (marine), the Baltic Sea (brackish) and landlocked lakes (freshwater); and they represent different susceptibility levels to the parasite *G. salaris* (Table 1).

**Table 1:** Population information: regional grouping, *G. salaris*-induced mortality level, salinity of the basin river flows to, mean water temperature, number of individuals (N), average call rate per population (CR), gene diversity (GD) and observed heterozygosity (*H_O_*).

Basin	Population	Population abbreviation	Coordinates		Mortality (%)	Salinity (July, ‰)	Mean water temperature (July, °C)	N	CR	GD	*H_O_*
**Landlocked**											
Lake Ladoga	Syskyanjoki	LL_SYS	61°38'N	31°16'E	0*	0	12†	32	0.999	0.208	0.218
Lake Onega	Pyalma	LL_PYA	62°24'N	35°52'E	0*	0	12†	40	0.998	0.176	0.181
V. Kuito	Pistojoki	LL_PIS	65°15'N	30°34'E	0*	0	12†	40	0.997	0.181	0.189
**Anadromous Baltic Sea**											
Gulf of Finland	Kunda	BA_KUN	59°31'N	26°32'E	10*	5**'**	17,1** ˘**	40	0.999	0.245	0.252
Gulf of Bothnia	Tornionjok	BA_TOR	65°49'N	24°9'E	10*	3**'**	16,1** ˘**	40	0.998	0.272	0.273
Gulf of Bothnia	Vindelälven	BA_VIN	63°44'N	20°19'E	10*	5**'**	14,4** ˘**	40	0.998	0.267	0.270
**Anadromous Atlantic Ocean**											
Barents Sea	Tuloma	B_TUL	68°53'N	33°0'E	98**	35^∼^	7,5^ ˘^	40	0.996	0.356	0.355
Barents Sea	Lebyazhya (Ponoi)	B_LEB	67°3'N	38°34'E	98**	35^∼^	7,5^ ˘^	40	0.998	0.340	0.345
Barents&White Sea	Yapoma (Varzuga)	BW_YAP	66°35'N	36° 9'E	98**	25**''**	15**''**	40	0.998	0.327	0.329
White Sea	Emtsa (S.Dvina)	W_EMT	63°32'N	41°52'E	98**	25**''**	15**''**	40	0.999	0.316	0.314
White Sea	Suma	W_SUM	64°17'N	35°24'E	98**	25**''**	15**''**	40	0.996	0.275	0.293
White Sea	Pon'goma	W_PON	65°20'N	34°24'E	98**	25**''**	15**''**	40	0.999	0.316	0.325

* *G.salaris* induced mortality level in the Atlantic salmon population. Based on (Kuusela *et al.* 2003, Bakke *et al.* 2002, 2004).

** Extrapolated from data of population from the Keret’ river, the White Sea basin (Kudersky *et al.* 2003) and Norwegian rivers (Johnsen & Jensen 1991), which have been almost wiped out after introduction of the parasite.

**'** http://www.itameriportaali.fi/en/tietoa/veden_liikkeet/en_GB/hydrografia/.

**''** http://www.itameriportaali.fi/en/muut_meret/en_GB/the_white_sea/.

**∼** http://www.nodc.noaa.gov/OC5/barsea/barmap.html.

**˘** http://water.travel.org.ua.

† http://www.nodc.noaa.gov/cgi-bin/OC5/SELECT/woaselect.pl.

### Sample preparation, SNP genotyping and data filtering

The SNP data analysed here are a sub-set of data that have been described previously in Bourret *et al*. ([37], Dryad accession number doi: 10.5061/dryad.gm367). Briefly, DNA was extracted from fin tissue using NucleoSpin Tissue (Macherey Nagel) columns, a salt-based protocol [49], or using vacuum extraction with glass beads (as in [50], with slight modifications). Salmon individuals were genotyped using an Atlantic salmon Illumina iSelect SNP chip [38] that successfully assayed 6176 SNPs. The number of SNPs per chromosome varied from 46 (chromosome 8) to 333 (chromosome 1), averaging 160. SNP genotyping and quality control procedures were as in [37]. We conducted additional filtering of the data using PLINK 1.07 [51] to eliminate SNPs with >10% missing data (2 SNPs), and SNPs exhibiting a minor allele frequency <0.05 across all populations (1543 SNPs); after filtering 4631 SNPs from 29 linkage groups remained. The existing female Atlantic salmon linkage map [38], which is 2127 cM in length, was used for assigning SNP map locations. The relative levels of population genetic diversity and divergence estimated using the SNP chip are well in line with estimates based on microsatellites in the same populations [47], [52] and therefore it is unlikely that ascertainment bias has had a large effect.

### Population genetics statistics

Basic population genetic parameters were calculated using PowerMarker 3.25 [53] and Arlequin 3.5 [54]. Analysis of molecular variance (AMOVA) was used to partition the within-population, between-population and between-group components of genetic variation and was also performed using Arlequin 3.5.

### Detecting loci under selection

**Figure 2:**
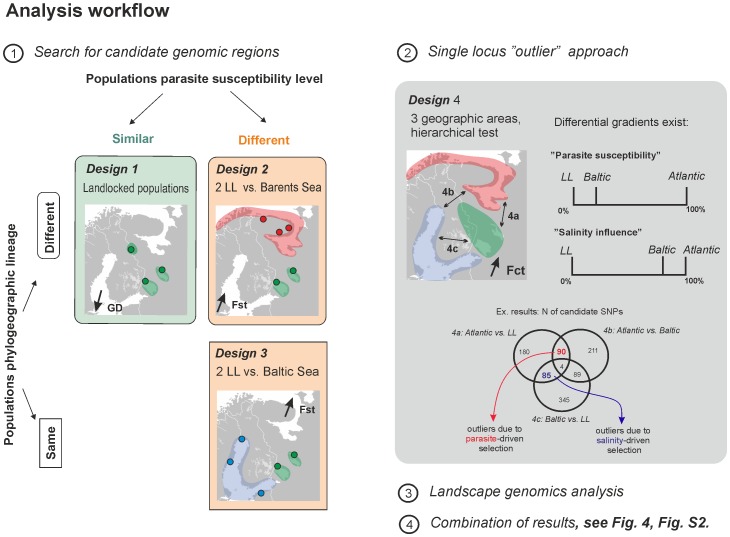
Analysis workflow and implementation of each of the methodological designs used.

As noted above, distinguishing between the molecular signatures of genetic drift and natural selection, and subsequent separation of the signatures of parasite-driven selection from selection acting upon other environmental factors is not straightforward. We therefore developed a novel analysis approach based on multiple tests for selection involving several combinations of populations that vary in geographic location and susceptibility to the parasite. These multiple tests hereafter will be referred as “designs” (summarised in Table 2, Figure 2). We assume that the strongest selective force after *G. salaris* presence is salinity of the basin fish migrates to [13]. By identifying loci detected as outliers in several analysis designs, i.e., overlapping loci, we aimed to avoid the detection of false positives and to pinpoint genomic regions that have a higher probability of being affected by either parasite-driven or salinity-driven selection.

**Table 2:** Methodological designs summary: geographic regions and populations involved, criteria of population choice (*G. salaris* susceptibility, salinity of the basin, phylogeographic lineage), number and percentage of candidate regions or “outlier” SNPs detected by each design.

Design	Populations/Regions	Comparisons	Susceptibility	Salinity	Lineage	Search target	N total^1^	N overlap^2^	N in final (Par, Sal) sets ^3^
**Kernel-smoothing based analysis**									
**1**	landlocked	LL_PIS vs. LL_PYA	similar, low	the same	different	reduced GD	27	6 (22.2%)	5 (18.5%)
		LL_PIS vs. LL_SYS							
		LL_SYS vs. LL_PYA							
**2**	Ladoga,Onega vs Barents	LL_SYS, LL_PYA vs. B_TUL	contrast	contrast	different	elevated *F*ST	7	5 (71%)	5 (71%)
		LL_SYS, LL_PYA vs. BW_YAP							
		LL_SYS, LL_PYA vs. B_LEB							
**3**	Ladoga,Onega vs Baltic	LL_SYS, LL_PYA vs. B_TOR	similar, moderate	contrast	same	elevated *F*ST	15	5 (33%)	4 (27%)
		LL_SYS, LL_PYA vs. B_VIN							
		LL_SYS, LL_PYA vs. B_KUN							
**Single outlier test based analysis**									
**4a**	Atlantic vs landlocked		contrast	contrast	mixed	elevated *F*CT	359 (7.7%)	28 (7.8%)	14 (3.9%)
**4b**	Atlantic vs Baltic		contrast	similar	different	elevated *F*CT	394 (8.5%)	21 (5.3%)	7 (1.7%)
**4c**	landlocked vs Baltic		similar, moderate	contrast	same	elevated *F*CT	523 (11.3%)	35 (6.7%)	7 (1.3%)

**1- N total**: total number of candidate regions or number of “outlier” SNPs identified by the design (with % of a total 4631 SNPs).

**2- N overlap**: number of candidate regions, overlapping with designs 1 – 3; or number of “outlier” SNPs falling into regions of overlap; (with % of N total).

**3- N in final sets**: number of candidate regions contributing to “Par” (parasite -affected) and “Sal” (salinity-affected) regions; number of “outlier” SNPs determining these regions; (with % of N total).

#### Designs 1 – 3: Candidate genome region approach

We first identified groups of adjacent markers that deviated from the chromosome-wide average levels of divergence (*F*_ST_) or diversity (GD) and hence represent genomic regions potentially subjected to natural selection [46]. SNP locus map positions were taken from [38], and analyses generally followed those outlined in Hohenlohe *et al.* [43] and Vaysse *et al.* [44]. More specifically, locus-specific *F*_ST_ values were first calculated using the approach described by Weir and Cockerham [55] and implemented in Arlequin 3.5. Locus-specific genetic diversity (GD) was estimated as expected heterozygosity using PowerMarker 3.25 software. To generate a smooth chromosome-wide distribution of the statistics, we used a kernel-smoothing moving average, as applied in the “locpoly” function included in the KernSmooth R-package [56]. For each genomic region centred on a certain centiMorgan (cM) position, the contribution of the *F*_ST_ or GD statistics to the regional average was estimated using local polynomials and a bandwidth of 3.5 cM (the half-length of estimated linkage disequilibrium in the dataset). The width proved to be appropriate to reduce sampling variance while being small enough to detect narrow regions of differentiation (data not shown). A permutation procedure (with 10,000 permutations) was performed to statistically test whether the smoothed curve was significantly (P ≤ 0.01) higher or lower than expected by chance within a local genome region. This candidate genomic region approach was applied to the following population comparisons.

Design 1 focused on completely *Gyrodactylus*-resistant landlocked populations (LL_PIS, LL_PYA, LL_SYS). Despite the very low genetic diversity in these populations (e.g. [36], [47]), regions with significantly reduced genetic diversity between populations may be the result of selection, as explored in the study of domestic dog breeds [44] and therefore identification of genomics regions with significantly reduced genetic diversity may be a fruitful approach for detecting signals of selection. The genetic diversity distribution, based on expected heterozygosity per SNP, was assessed in all three populations using the kernel smoothing approach outlined above, and when reduced GD was observed in at least one population the region was considered to be a candidate that is potentially under directional selection.

Designs 2 and 3 focused on comparisons between two landlocked lakes (Ladoga and Onega) and either populations from the Barents Sea **(design 2**) or from the Baltic Sea (**design 3**). When compared to populations from landlocked lakes, Barents and Baltic Sea salmon populations differ in their phylogeographic history and vary in their relative response to the parasite, salinity of the basin and, possibly, other environmental characteristics; therefore these designs may help to disentangle the effects. The landlocked population, V. Kuito lake (LL_PIS), was excluded from these designs as it originates from a different glacial refugium than the other landlocked populations [36], [37]. For both design 2 and 3 we identified regions with elevated *F*_ST_ by conducting six pair-wise population comparisons: **design 2)** between Ladoga (LL_SYS) & Onega (LL_PYA) and three Barents Sea populations (B_TUL, B_LEB, BW_YAP), and **design 3**) between Ladoga (LL_SYS) & Onega (LL_PYA) and Baltic Sea populations (BA_TOR, BA_VIN, BA_KUN). In each case, regions were identified as potentially under directional selection when elevated *F*_ST_ was observed in a particular genomic region for at least two comparisons out of six, with the additional criterion that both LL_SYS and LL_PYA should be represented in the significant comparisons. This ensured that observed elevations in *F*_ST_ statistics were not artefacts of a high genetic drift in a single landlocked population. Populations from the White Sea were not included in Design 2 in order to avoid a skew in number of individual tests between designs 2 and 3.

#### Design 4. Single-outlier test

To detect the potential effects of selection on a regional scale, we applied an *F*_CT_ - based “outlier” test that accounts for hierarchical population structure [41], as implemented in Arlequin 3.5. Outlier loci are detected based on differentiation among groups of populations (*F*_CT_), controlled for heterozygosity of the loci. The expected distribution of *F*_CT_ values was obtained by 50,000 coalescent simulations, and loci falling outside the upper boundary of the 0.95 confidence interval were considered to be potentially affected by directional selection.

The hierarchical structure was formed based on the *G. salaris* susceptibility levels. Populations were combined into three groups: susceptible Atlantic Ocean (B_TUL, B_LEB, BW_YAP, W_EMT, W_SUM, W_PON), tolerant Baltic Sea (BA_TOR, BA_VIN, BA_KUN), and resistant landlocked populations (LL_PIS, LL_PYA, LL_SYS). Three pair-wise outlier tests were performed: **design** **4a**) Atlantic group vs. landlocked, **4b**) Atlantic vs. Baltic, **4c**) Baltic vs. landlocked. Resulting single SNPs outliers could further be separated into a category of SNPs influenced by parasite and another category of SNPs influenced by salinity. To do so we applied the following logic. Populations from the Atlantic group are completely susceptible to *G. salaris*, whereas populations from the Baltic and landlocked groups are, to some extent, similar in their response to the parasite: with a certain level of effectiveness, they are able to tolerate the pathogen [30]. Therefore, outliers common for both **4a** (Atlantic group vs. landlocked) and **4b** (Atlantic vs. Baltic) designs, but not found in **4c** (Baltic vs. landlocked), are considered more likely to be the result of parasite-driven selection occurring in landlocked and Baltic populations. Likewise, the Atlantic and Baltic groups both migrate to saline environments (marine and brackish water), whereas landlocked populations never experience a saline environment as they migrate to freshwater lakes. Thus, we predict that outliers common for both **4a** (Atlantic group vs. landlocked) and **4c** (Baltic vs. landlocked) designs, but not for **4b** (Atlantic vs. Baltic), are more likely to be affected by salinity-driven selection in the Atlantic and Baltic populations compared to the landlocked populations.

#### Combining information from several designs

The genomic regions in which “footprints” of selection were detected with each of designs 1–3 were compared identify overlaps in selection footprints between the designs. Cases where the same region was detected by at least two of the three designs were highlighted as promising candidates that are potentially affected by some form of selection. We then plotted outlier SNPs detected in design 4 along the genome to relate the linkage map position of these loci to the regions detected by designs 1 – 3. Genomic regions highlighted as promising candidates potentially affected by some form of selection in designs 1 – 3 and that also included “Parasite-influenced” outliers (common outliers from designs 4a and 4b) were considered promising candidate regions that are potentially affected by parasite-driven selection. The same logic was applied for “Salinity-influenced” outliers (common outliers from designs 4a and 4c) to identify genomic regions harbouring signals consistent with salinity-driven selection.

### Landscape genomics analysis

The methodological designs described above aim to target parasite-driven selection by separating the “parasite” effect from environmental effects, of which we assume salinity to be one of the most prominent. To additionally test for associations between SNP allele frequencies and other varying environmental characteristics, the landscape genomics approach of Frichot *et al.* ([57]) implemented in LFMM (“latent factor mixed models”) software, was applied. This method uses a hierarchical Bayesian mixed model based on a variant of principal component analysis, and accounts for residual population structure which is introduced via unobserved or latent factors. The LFMM algorithm efficiently estimates random effects due to population history and isolation-by-distance, and does not require a control data set of *a priori* neutral loci [57]. Genetic-environment correlation was tested for the whole set of 4631 SNPs; environmental variables included population coordinates and mean surface water temperature, in addition to estimates of *G. salaris* - induced mortality rate and surface salinity of the basin. The number of latent factors required for LFMM computations, which is the number of clusters best describing structure of the original data, was set to 12. It equals to the number of original populations in our dataset, which were shown to be genetically distinct from each other previously [36], [37], [52]. Absence of within-population structure was additionally tested using STRUCTURE.2.3.4 [58] software. Bonferroni correction for multiple tests was applied to significant SNPs.

### SNP annotation and gene ontology enrichment analysis

Annotation of all 4631 SNPs to specific gene ontology (GO) terms was performed using a customised python-based procedure intended to maximise the number of successfully annotated markers. Briefly, the general steps included tblastx and blastx searches of SNPs flanking sequences against nucleotide and protein NCBI databases with a 1×10^-10^ e-value threshold followed by subsequent collection of corresponding human GO identifiers (as of 03.04.2012) from the GO database (www.geneontology.org). For further enrichment and functional analysis, a more recent version of the GO database (as of 08.10.2013) was used.

Enrichment analyses could only be conducted on the results of tests that identified single SNP outliers, as opposed to genomic regions i.e. design 4 and landscape genomics analysis. To determine whether sets of parasite- and salinity-affected single SNP outliers identified by design 4 and by landscape genomics analysis were significantly enriched or depleted for particular GO terms, a hypergeometric test for GO term over- and under-representation was performed in Cytoscape 2.8.3. [59] using the BiNGO 2.44 [60] plugin. For design 4, 58 GO terms associated with annotated parasite-affected outliers and 47 GO terms associated with annotated salinity-affected SNPs were investigated. For landscape genomics analysis, the respective sets included 318 and 116 GO terms. As a reference set during the analysis we used the annotations for all 2857 SNPs, for which any annotation was available using the procedure outlined above. A significance level of 0.05 and the Benjamini & Hochberg false discovery rate correction were applied. The same enrichment/depletion analysis was performed using a version of GO database with reduced redundancy (Generic GO slim, 09.10.2013), in order to obtain an additional generalized overview of possible functions “shared” by the studied GO terms and respective SNP outliers.

Functional relatedness of GO terms included in the “parasite” and “salinity” lists of outliers resulting both from design 4 and landscape genomics analysis was assessed using the Cytoscape plugin ClueGo1.7.1 [61], which constructs and compares networks of functionally related GO terms, using human gene ontology (08.10.2013). A two-sided hypergeometric test (enrichment/depletion) was applied, network specificity was set to ‘medium’(as in [62]), and false discovery rate correction was performed as above.

## Results

### Population genetic structure

Basic genetic statistics across populations and markers are presented in Table 1, and locus-specific information is presented in Table S1. Allele frequencies per SNP per population are presented in File S1. As expected, landlocked populations were characterised by the lowest values of observed heterozygosity (from 0.181 LL_PYA to 0.218 LL_SYS) and genetic diversity (from 0.176 LL_PYA to 0.209 LL_SYS). Anadromous Baltic Sea populations had intermediate values of *H*_O_ and GD (0.252 and 0.245 in BA_KUN to 0.273 and 0.272 in BA_TOR, respectively), while populations from the Barents and White Seas were the most variable (from 0.293 and 0.275 (BW_SUM) to 0.355 and 0.356 (BW_TUL), respectively; Table 2).

All pair-wise comparisons showed highly significant levels of population genetic differentiation (P<10^-5^, Table S2). The highest *F_ST_* values were observed within landlocked comparisons (from 0.32 LL_PYA vs. LL_SYS to 0.52 for LL_PYA vs. LL_PIS); Baltic Sea comparisons varied between 0.08 (BA_TOR vs. BA_VIN) to 0.23 (BA_VIN vs. BA_KUN), while the Barents and White Sea populations were the least differentiated: *F_ST_* varied from 0.05 (BW_LEB vs. BW_TUL) to 0.18 (BW_SUM vs. BW_EMT).

An AMOVA analysis indicated a significant genetic variation among and within geographic regions (Atlantic Ocean, Baltic Sea, landlocked populations). Notably, the amount of genetic variation explained by the within-region component (17.8%, P<< 0.01) is higher than the variation explained by the among-region component (9.6%, P<<0.01), indicating markedly high between-population differentiation within regions (Table S3).

### Signatures of directional selection

#### Designs 1–3: Candidate genomic regions under selection

**Figure 3:**
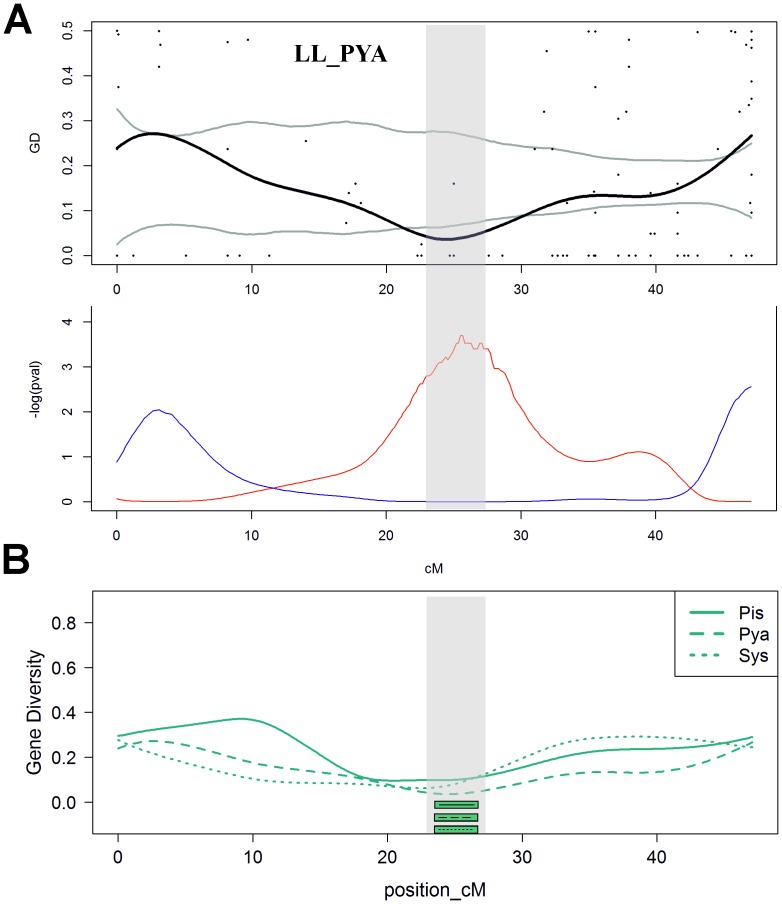
Example of the results of a candidate region analysis (chromosome 23, design 1): search for regions of reduced genetic diversity in landlocked populations. A. One of the three populations, LL_PYA. SNP *GD* values (black dots), kernel-smoothed distribution of GD (black line) along the chromosome and the 99% confidence interval (area within grey lines) are shown. Distributions of logarithmically scaled p-values for elevated (blue) and reduced (red) GD statistic are plotted below. B. Smoothed *GD* curves for all three populations. Horizontal bars indicate regions of significant (p≤0.01) reduction of GD. Vertical grey shading represents region which is significant in all three populations and which has been considered further as one of a candidate regions under selection, detected by design 1.

**Figure 4:**
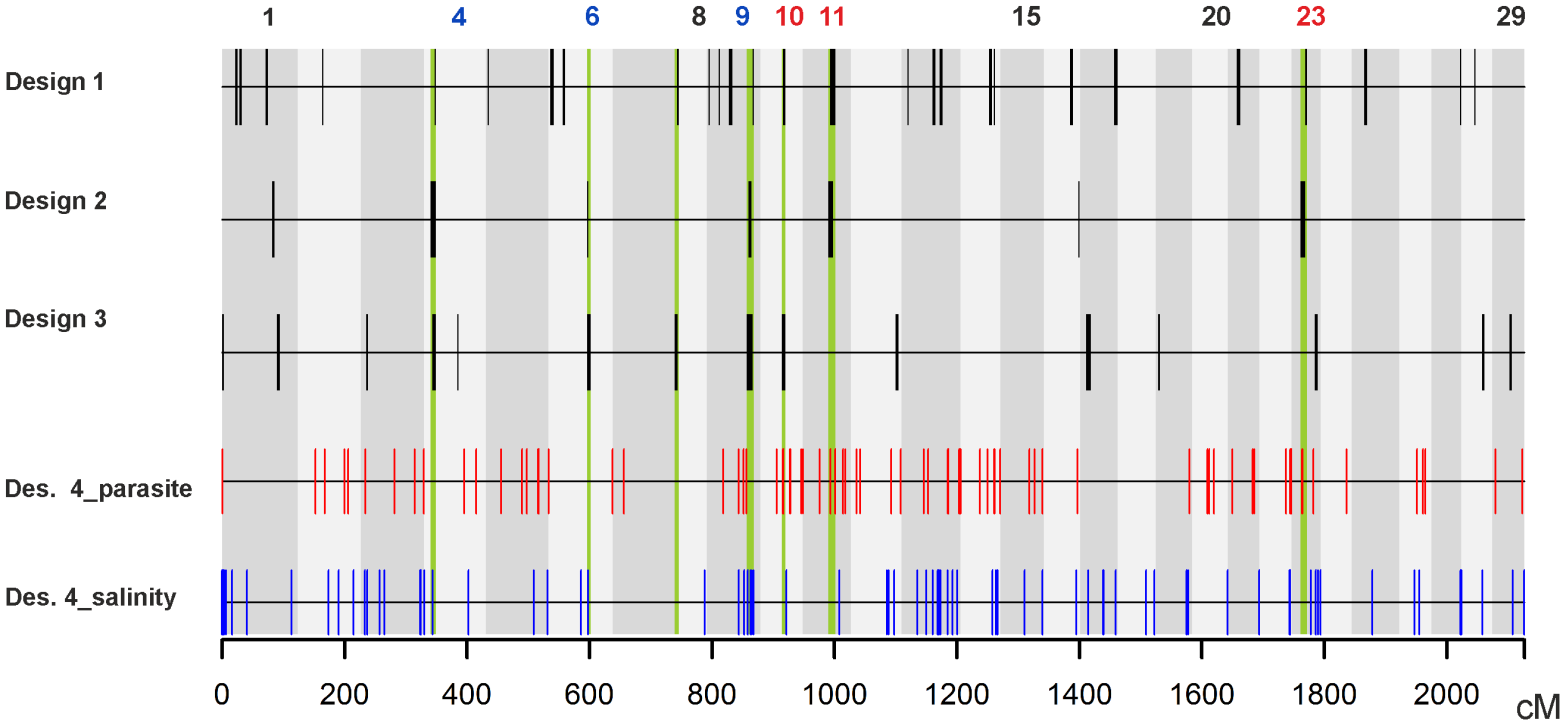
Final overlap of results based on all applied designs: genome-wide evidence of directional selection. Vertical coloured shadings (green) show genomic regions, where two or three regions detected by kernel-smoothing-based designs overlap. “Parasite” (red) and “salinity” (blue) single outlier SNPs from design 4 are plotted as smaller vertical lines. Chromosome numbers are given, chromosomes bearing regions exclusively containing “parasite outliers” are marked with red font colour, and “salinity outliers” - with blue.

Regarding reductions in gene diversity within the landlocked populations (design **1:** see Figure 3 for a single population example), 27 regions across 18 chromosomes had significantly reduced GD (Table 2, S4, S5; Figure 4). Comparisons of lakes Ladoga and Onega versus three populations from Barents Sea (design **2**) or Baltic Sea (design **3**) exhibited significant differentiation at 7 regions across 7 chromosomes and 15 regions from 13 chromosomes, respectively.

#### Design 4: Single locus selection test

Hierarchical genome scans were performed in a pair-wise manner between geographic regions to detect markers that are potentially under directional selection. In total, 359 SNPs (7.7%) showed signals of selection when comparing Atlantic vs. landlocked populations (design **4a**), 394 (8.5%) in the Atlantic vs. Baltic comparison (design **4b**), and 523 (11.3%) in the Baltic vs. landlocked test (design **4c**) (Figure 2_2). Using the approach outlined in the Methods, 90 (1.9%) and 85 (1.8%) outliers were found consistent with parasite- and salinity-driven selection, respectively. (Table S4, Figure 4).

#### Combined signals of selection

Results of all comparisons in designs 1 – 3 were combined to identify genomic regions suggested to be potentially affected by selection by at least two of the three designs. In total, seven genomic regions were identified (Figure 4): five regions in two comparisons out of three and two regions in all three comparisons. We then identified specific loci within these seven genomic regions that were also indicated to be affected by either parasite- or salinity-driven selection in the single locus selection tests (design 4). Three genomic regions potentially affected by parasite- but not salinity-driven selection were identified (chromosomes 10, 11, 23), and another three regions potentially affected by salinity- but not parasite-driven selection were also identified (chromosomes 4, 6, 9) based on combination of signals from designs 1 – 4 (Tables 3, S4, S6; Figure 4). Therefore, a total of six genomic regions were shown to be affected by either parasite- or salinity- influenced selection, based on designs 1 – 4. Along with parasite- and salinity- influenced outliers, design 4 has identified a number of SNPs possibly affected by other forms of directional selection.

**Table 3:** Contribution of each methodological design to the final sets of overlapping candidate regions, as marked with “x”.

Candidate regions		Designs		
Selection force	Chromosome	1	2	3
Parasite	10	x		x
	11	x	x	
	23	x	x	
Salinity	4	x	x	x
	6		x	x
	9	x	x	x

### Landscape genomics analysis

The number of SNPs significantly correlated with environmental characteristics varied considerably among the environmental variables. The greatest number of SNPs (523) was correlated with *G. salaris-*induced mortality, followed by the SNPs correlated with longitude (193 SNPs), salinity of the basin (179 SNPs), latitude (10 SNPs), and water temperature (6 SNPs). The same SNPs were frequently detected to be correlated with several environmental characteristics, with salinity and *G. salaris-*induced mortality associated with the same SNP on 124 occasions (Table 4, Figure S1, Figure S2). Detailed information about significant SNPs is presented in Table S4.

**Table 4:** Results of the landscape genomics analysis (LFMM).

Environmental characteristics	N of outliers	N of unique outliers
Latitude	10 (0.2%)	5 (50%)
Longitude	193 (4.2%)	123 (63.7%)
Mean water temperature (July, °C)	6 (0.1%)	2 (33.3%)
Mortality (%)	523 (11.3%)	328 (62.7%)
Salinity (July, ‰)	179 (3.9%)	22 (12.3%)

**N of outliers**: number (and % from the total) of SNPs correlated with environmental characteristics.

**N of unique outliers**: subset (and % from N of outliers) of SNPs which are correlated only with a given characteristic.

### Annotation of outliers

A total of 2857 SNPs were successfully annotated (GO database as of 05.04.2013) and used further for the enrichment tests (Table S7). For design 4 (Arlequin3.5-based test), these included 58 of 90 identified parasite-affected outliers and 47 of 85 salinity affected outliers. For landscape genomics analysis, 318 SNPs of the 523 parasite-correlated and 116 of the 179 salinity-correlated SNPs were annotated (Table S8). For both the outliers identified with design 4, and with the landscape genomic analysis, no significantly over- or under-represented GO terms were detected after correction for multiple testing using the complete GO database (as of 08.10.2010), nor when using a reduced redundancy GO_slim database (as of 09.10.2013) (Tables S9, S10).

Functional network analysis conducted on outliers identified by design 4 however, grouped SNPs in the parasite- or salinity-driven selection sets into three significant and non-overlapping functional clusters (Table S11a). Terms in the first cluster included GO terms associated exclusively with the salinity-driven selection SNP set and were involved in functional processes associated with negative regulation of the protein kinase B signalling cascade and renal absorption. Clusters 2 and 3 were comprised solely of SNPs from the parasite-driven selection SNP set and were functionally involved in translation pre-initiation (cluster 2) and long-chain fatty-acyl-CoA metabolism (cluster 3). GO terms associated with significant SNPs from the landscape genomics analysis also divided into clusters composed of parasite- and salinity- related terms, though the number of actual functional processes each of the terms was part of was much higher, most likely due to the large number of SNPs in the analysis, making the results for these loci difficult to interpret (Table S11b).

## Discussion

The goal of this study was to reveal the genetic basis of Atlantic salmon inter-population susceptibility differences to the parasite *Gyrodactulus salaris*. We have identified a number of single SNP markers and several regions in the Atlantic salmon genome exhibiting signatures of directional selection. Based on the comparison and combination of results from different tests and methodological designs, we propose at least three genomic regions, on chromosomes 10, 11 and 23, potentially contributing to parasite resistance in Atlantic salmon and three regions, on chromosomes 4, 6, 9, presumably influenced by salinity-driven selection.

The combination of the results of several tests has been applied in a number of studies in order to strengthen the reliability of the identification of genome regions that are potentially affected by selection (e.g. [45], [63]–[65]). The suggested advantage of such an approach is that the final results are based on the combination of several lines of evidence, and hence, the probability of Type I error is reduced. In other words, different lines of evidence showing the same (parallel) signal of selection are unlikely to be caused by drift. In our study we used the existing female Atlantic salmon linkage map [38], which is 2127 cM in length, to estimate how many regions consistent with directional selection we would expect to identify based on chance alone for a single test. Given that the bandwidth we used for candidate-region kernel-smoothing detection was 3.5 cM, the whole genome can be divided into 607 3.5 cM regions. The probability of any given region being identified as an outlier purely by chance is equal to the significance threshold used in the analyses, i.e., 0.01. Therefore, on average, 6.07 regions per test are expected to be the result of a Type I error. The number of candidate regions detected by single designs was seven in design **2**, 15 in design **3**, and 27 in design **1**. Therefore, at least designs **1** and **3** are likely to have identified a number of regions potentially affected by selection. The likelihood of identifying the same genomic region in two tests purely by chance is likely to be considerably lower. However, as the designs implemented in this study are not completely independent it is not possible to make an exact calculation. Given that the same landlocked populations (lakes Ladoga and Onega) were included in all three designs, specific genetic features of one or more of those populations could potentially result in an increase in the detection of (false) positives in more than one design for this reason, rather than due to selection. On the other hand, as the landlocked populations are the only populations known to be totally resistant to *G. salaris*, there are no clear alternatives. That said, we attempted to limit the potentially biasing effects of a single landlocked population by only including outlier signals detected in more than one landlocked population in designs 2 and 3 (Tables S5, S6). Moreover, even though it should be treated with caution [66], the functional network analysis provides further support to the detected separation of parasite- and salinity-affected candidate regions (see below). Nevertheless, as highlighted in earlier studies [63], [65], while support of multiple tests elevates the candidate status of the loci/region, further analysis is required to determine the role of the identified genomic regions in potential selective processes.

As outlined in Methods, an assignment of the seven regions detected with multiple designs to either parasite-or salinity-affected modes of selection was based on results of the single outlier tests (design **4**). It has been proposed, however, that *F_ST_* -based outliers might not only be an outcome of natural selection but can also indicate a presence of intrinsic barriers of gene flow, especially when the existence of intrinsically incompatible alleles is coupled with environmental or ecological constraints of gene flow [67]. This could also be the case in our study, resulting in some of the detected selection-affected outliers being “false positives”. Design 1, however, is based on the assumption of reduced population gene diversity, rather than increased divergence, which allows us to strengthen the candidacy of detected outliers: single *F_CT_* -based outliers (Table S4) falling within the detected regions are likely to be affected by directional selection. It can be argued, however, that some of genomic regions and SNP outliers detected by designs 1 – 4 can be artefacts of non-selective forces such as population history and e.g. reduced recombination [68]. Since all significant regions identified with designs 2 – 4 comprise several populations we consider them to be relatively robust to the abovementioned caveats, however genome regions with low intra-population diversity detected by design 1 may be more likely to be affected by non-selective forces. However, little is currently known about the recombination rate differences in fish genomes [69] and therefore more thorough scrutiny of the reduced diversity test awaits further research.

The fact that the candidate loci/regions were based on the results of several tests may also explain the differences in our results compared to those recently reported by Bourret *et al.* [37], who conducted several tests to identify signals of parallel adaptation in Atlantic salmon populations spanning their natural distribution. Only one of the seven final candidate genomic regions detected in our study (chr 11) was also identified by Bourret *et al.*, although several regions detected by only one of the designs were described in the mentioned study. This could be due to the more stringent criteria of our testing design and/or because Bourret *et al*. included populations from across the whole European distribution of the species in most outlier tests and were thus looking for more general trends. More detailed comparisons would be required to distinguish between these alternatives.

### Parasite-driven selection

At least three genomic regions affected by parasite-driven selection have been identified in our study. Given the plausible implication of these regions in the mechanisms of immune response, our findings support the existing conception of polygenic control of immunity in Atlantic salmon. Differential expression studies in response to various pathogens including *Aeromonas salmonicida* and *G. salaris* identified significant RNA expression changes in up to 162 transcripts [70], [71]. A subsequent assessment of microsatellite loci identified in the untranslated regions of some of these transcripts detected signals of selection in a number of loci [13]. Although the associated markers were assigned to particular linkage groups in some of the studies [21], the exact position of the candidate loci is generally unknown. A QTL approach has also earlier identified multiple genomic regions associated with *G. salaris*-tolerance in Atlantic salmon involving a back-cross of Scottish (*G. salaris* susceptible) and Baltic (*G. salaris* tolerant) Atlantic salmon [72]. However, the identified genetic regions associated with *G. salaris* resistance were different to those highlighted by our study. Marker-trait associations in the Gilbey *et al.* [72] study represented entire linkage groups [73]; thus, a direct comparison of results is difficult. Furthermore, the fact that our study also included resistant landlocked populations could also explain the differences in results. Additionally, our study focused on detecting signals of exclusively directional selection because *F*_ST_-based methods have a number of limitations if applied to search for signals of balancing selection, including high rates of false positives [41], the tendency to underestimate the differentiation of polymorphic loci [74] and low power when using simulated data sets [75]. Balancing selection, however, is thought to be an important mechanism that maintains the diversity of immune system-related loci such as those of the major histocompatibility complex (MHC; [2], [76]–[78]). Therefore, some immune-related loci, such as MHC genes, are not expected to be detected by our analysis, in contrast to a QTL approach. Nevertheless, MHC loci and immune-related genes can also be the target of directional selection [79], [80]. Therefore, given that pathogen presence can cause strong directional selection [1], [81], the fact that some immune loci under balancing selection might have not been identified by our analysis does not conflict with the primary aim of the study to detect specific loci contributing to *G. salaris* resistance in Atlantic salmon.

Functional network analysis of the design 4 “parasite” influenced outliers is consistent with an immune-related role for these regions. However, caution is warranted because it is possible that the observed signals of selection may be due to selection acting on a tightly linked gene with a different function. Recently, the potential dangers of ‘storytelling’ based on GO annotations has also been cautioned [66]. Nevertheless, when applied responsibly, it has been demonstrated that the GO can provide valuable functional information in species with poorly annotated genomes, such as salmonid fishes [82]. “Parasite-associated” GO terms resulting from design 4 clustered in two functional groups (Table S11a). The first group was united by two genes involved in translation initiation, EIF3B and EIF3D (eukaryotic translation initiation factors). IEF3 genes have been suggested to contribute to increased translation rates during T lymphocyte activation [83], [84], as well as to play role in immunoreceptor signal transduction [85], and early stages of HIV [86] and hepatitis C [87] infection. The mentioned studies are human-based, yet immune system of fish have analogues of T cells, and functional similarities between immune system of fish and higher vertebrates have been strongly suggested [88]. More generally, EIF3 translation initiation factors contribute to translational control of a variety of stress responses in addition to pathogen challenge, including osmotic stress, nutrient starvation, temperature stress (rev. by [89]). The system is considered to be very conserved (rev. by [90]) and is observed in a variety of organisms including green alga [91], plants [92], yeast [93] and mammals [94], therefore its precise role in Atlantic salmon populations studied here is still open for discussion. The second functional group of design 4 - based “parasite” GO terms were united by two genes involved in fatty acids synthesis and elongation, FASN (fatty acid synthase) and HSD17B12 (estradiol dehydrogenase). A substantial number of studies on a range of mammals have shown that fatty acids, especially n-3 and n-6 polyunsaturated fatty acids (PUFA), play a crucial role in the formation of immune response; in experimental studies, over-supplied PUFAs performed as anti-infection, anti-malaria, anti-tuberculosis, and anti-fungal agents (rev. by [95]–[97]). Balance between the n-3 and n-6 PUFAs is essential for the regulation of inflammation and the functioning of immune cells [98], [99]. For instance, an excess of n-3 PUFAs leads to reductions of secreted interleukin-1β (IL- 1β), interferon-α (IFN-α), and a number of other cytokines, which subsequently lead to decreased inflammation [100], [101]. Interestingly, the dissimilarity in cytokines produced in the first stage of inflammation was shown to be the key difference in the way Atlantic salmon with different susceptibility respond to *G. salaris* [102]. An experimental study demonstrated that highly susceptible salmon from the east Atlantic responded to *G. salaris* exposure by enhanced production of IL- 1β and IFN-γ cytokines and elevated proliferation of inflammatory cells, which, in turn, triggers the proliferation of epithelial and mucous cells that the parasite feeds on. Therefore, susceptible salmon launch an immune response that might actually be beneficial to the parasite [102], [103]. Conversely, tolerant Baltic Sea salmon respond to *G. salaris* infection later in time and with up-regulation of genes encoding other molecules: IFN-γ, T-cell receptor-α and serum amyloid A [102]. Given the property of n-3 fatty acids to reduce a secretion of pro-inflammatory IL-1β, their activity might be especially beneficial during the early stages of *G. salaris*-mediated immune response, when, according to Kania *et al.* [102], decreased inflammation and mucus formation would have a negative impact on parasite survival. From this perspective, the fact that “parasite-associated“ GO terms clustered into a functional network united by fatty acid metabolism brings additional evidence of fatty acid involvement in immune response and supports the notion of a broad range of molecules being immune-relevant [12], [13]. We also consider the n-3 polyunsaturated fatty acids to be good candidates for agents underlying the effective immune response performed by tolerant Atlantic salmon in response to *G. salaris* infection. *G. salaris*, like other parasitic flatworms, ([104], Aisala & Lumme, in preparation) lacks the enzymes for *de novo* synthesis of the fatty acids, but has a collection of enzymes needed in transporting, lengthening and processing them; the dependency on the host for fatty acid synthesis might be a key to understanding the role of the above observations. However, acclimation to salinity was also shown to alter composition of PUFAs, e.g. in tilapia, *Oreochromis mossambicus,* [105], and two mullet species (*Chelon labrosus* and *Mugil cephalus*) [106], [107], which was suggested to be due to the substantial energetic demands preceding activation of acclimation processes [106]. Thus, it appears that studies addressing the role of lipid metabolism in terms of fish osmoregulation have not reached a consensus as the data are contradictory (rev. by [108]). Additionally, fatty acids composition in the cell membrane can affect its fluidity and consequently have an effect on velocity of Na+/K+ ion pumps, which is one of the mechanisms of acclimation to water temperature changes (rev. by [109]). But given that change in water temperatures does not necessarily lead to change in fatty acids composition [109], [110], the precise role of fatty acids in temperature acclimation remains unclear.

### Salinity-driven selection

Three genomic regions presumably affected by salinity-induced selection were identified in our study. To our knowledge, an outlier approach has not previously been used to investigate the genetic basis of salinity tolerance in Atlantic salmon. However, there have been a number of investigations of this topic in salmonids and other fish species using alternative approaches. The effect of varying salinity levels on the RNA expression level of genes known to be involved in osmoregulation has been studied in species with varying salinity tolerance [111]. At the intraspecific level, approaches for studying the genetic basis of salinity tolerance include differential RNA and protein expression analyses, as well as QTL studies. These studies have indicated that there are several important molecular components involved in salinity acclimation at the population level. These include Na^+^/K^+^ ATP-ase [112], [113], pathways involving thyroid hormones [114] and cortisol [115], as well as molecules involved in intracellular calcium levels, such as otopetrin 1 [114]. Altogether, more than 100 proteins have been shown to be differentially expressed in a recent study of fresh- and brackish-water spawning whitefish [116], emphasising the complexity of salinity-tolerance mechanisms. Along with salinity tolerance, it is tempting to speculate that smoltification related processes could be the target of differential selection in studied populations. Land-locked salmon have been shown to undergo the smoltification process even though there is no known physiological explanation for it [117]. Moreover, it has been suggested that the process of smoltification may in fact be a maladaption in freshwater salmon populations [117], being therefore a presumably strong selective force acting on them. Direct comparison of our results with those of RNA and protein expression studies is difficult due to the currently limited information regarding the specific genomic locations of the genes in question in the Atlantic salmon genome. However, a comparison with studies using a QTL approach is possible. One of the three regions identified in our study that is presumably affected by salinity-induced selection (the distal part of the q-arm of chromosome 9) was also identified by Norman *et al.,* [118], who used a QTL approach to pinpoint genomic regions involved in osmoregulation in several salmonid fishes, including Atlantic salmon. This region has been shown to include the calcium-sensor receptor, *CaSR* [118]. This receptor senses the extracellular Ca^+^ concentration and activates the appropriate intracellular signalling pathways, acting as an internal salinity sensor [119]. *CaSR* has been shown to be upregulated during smoltification in Atlantic salmon, supporting its importance in osmoregulation [120]. Interestingly, this observation is in strong concordance with the study of Papakostas *et al.,* [116], where proteins expressed differentially between fresh- and brackishwater whitefish in different salinities were shown to be enriched for Gene Ontology (GO) biological process terms, including calcium ion transport and calcium channel regulator activity. Thus, based on independent studies using a variety of approaches, *CaSR* appears to be a very strong candidate for involvement in salinity adaptation in salmonid fishes. Although the remaining two genomic regions we identified were not identified in the QTL study of Norman *et al.,* [118], this is perhaps not surprising given that North American Atlantic salmon were used in their study, as opposed to European populations in the current study, because the North American and European lineages are thought to have diverged more than one million years ago and include differences in chromosome number [35], [121], [122].

Functional network analysis suggested that the “salinity-selected” SNPs detected by design 4 were associated with GO biological process terms involved in the protein kinase B cascade and peptidyl-tyrosine phosphorylation (Table S11a), although, as mentioned above, this analysis has to be interpreted with some caution. The role of the protein kinase B (PKB) signalling pathway is not directly evident in relation to osmoregulation and smoltification given the broad range of processes the pathway is involved in [123]. Nevertheless, the PKB cascade was shown to be activated by hyperosmolarity stress [124], suggesting that it plays role in salinity-mediated stress signalling. When actual genes associated with the “salinity” group of GO terms are considered, osmoregulatory function was suggested, e.g., for the *Slc9a3r1* (sodium-hydrogen antiporter), which functions in kidney of mice and controls phosphate and uric acid concentration in urine output [125], [126]. Nonetheless, the very same gene was suggested to have immune functions as well: the protein it codes is psoriasis responsible and is implicated in immune synapse formation in T cells [127]. Notably, other genes associated with “salinity” group of GO terms also have roles in immune processes. RUVBL1 (contributes to ATP-ase activity) promotes activation of gene transcription and, when up-regulated, is likely to provoke functional enhancement of immune cells and the enhancement of the subsequent immune response involving B cells, T cells and macrophages [128]. *PPP2R1A* (serine/threonine-protein phosphatase regulatory subunit A) contributes to regulation of cell autophagy as part of an anti-bacterial response [129], and is involved in T lymphocytes signaling processes throughout porcine respiratory syndrome virus infection (rev. by [130]). These results suggest that presumably “salinity” - affected outliers, identified by the design 4, might also be due to population differences in response to *G. salaris*. The landscape genomic analysis additionally highlighted that distinguishing between selection acting upon different environmental factors is not straightforward (see below).

### Landscape genomics analysis

The analyses used in our study were designed to provide a means to tease apart the effects of pathogen driven selection and other potential selective forces. We considered a simple scenario where the only environmental characteristic, salinity of the basin fish migrates to, was taken into consideration. Nevertheless, we also tested for associations between SNPs allele frequencies and other varying environmental factors using the LFMM approach.

The number of significant SNPs varied greatly depending on the environmental characteristic in question. Unsurprisingly, the greatest number of SNPs (523, of which 328 unique) was correlated with mortality rate in presence of *G. salaris* (Figure S2). When compared to a set of “parasite”- influenced “outlier” SNPs derived from design 4, 32 out of 90 outliers were also present in the set of unique “mortality” LFMM-based SNPs; or 84 SNPs out of 90 if all 523 LFMM-outliers were considered. The greatest overlap (124 SNPs) was observed between “mortality” and “salinity” environmental characteristics (Figure S1), indicating that the effect of these factors cannot be disetangled using the landscape genomics approach in this study. Therefore, we did not utilize the “mortality” and “salinity” correlated SNPs in identifying the final genome regions affected by these selective forces. Nevertheless, they might additionally strengthen some of the detected regions, where unique LFMM-based outliers duplicate either “parasite” (chr 10, 23) or “salinity” (chr 6) outliers resulting from design 4.

A surprisingly low number of SNPs were correlated with water temperature (6, 2 unique i.e. only correlated with water temperature and no other variable), given that temperature regime is often associated with population genetic diversity in salmonids (rev. by [131]) and other fish species (e.g. herring, [132]). While adult Atlantic salmon show a great capacity of temperature acclimation [133], water temperature can affect population genetic structure through juvenile mortality, since survival of salmon juveniles greatly depends on water temperature regimes (rev. by [134]). And since in the current study (as in many others, e.g. [13]) we used mean sea/lake surface temperature, it is possible that we have not detected otherwise existing correlations with river temperature. The other possibility is that for salmon populations described in this paper, other environmental characteristics, such as landscape features of river systems, might play a more prominent role than water temperature [134]. Altogether however, lack of temperature-associated genetic variation is confirmed by similarly small number of SNPs correlated with latitude (10, 5 unique) (Figure S2), since water and air temperatures are closely correlated with latitude. Studies of Atlantic salmon in Canada, for example, showed that genetic diversity of MHC genes increases with temperature along a latitudinal gradient, which was suggested to be in response to pathogen selective pressure [8]. Somewhat unexpected however, is the substantial number of SNPs significantly correlated with longitude (193, 123 unique) (Figure S2).

## Conclusion

We identified at least three genomic regions potentially contributing to *G. salaris* resistance in Baltic Sea and landlocked freshwater Atlantic salmon, compared to susceptible salmon from the Atlantic Ocean. We detected three additional genomic regions exhibiting signatures of selection that are possibly salinity-related. These genomic regions are a good starting point for further research to identify the specific genes contributing to the signals of directional selection. For instance, targeted sequencing of detected regions might be a promising approach, especially given that the Atlantic salmon genome sequence is soon to be publically available [135]. Although the non-independence of tests remains an issue, our approach of outlier identification incorporating several outlier tests based on different assumptions provides encouraging results for overcoming challenges related to high population divergence and environmental selection pressures mimicking the influence of *G. salaris*. The validity of the separation of the effects of parasite-driven vs. salinity-driven selection was, to some extent, supported by a functional network analysis of GO terms associated with the detected outliers. Given the unlikeliness that the observed results could be explained by chance alone, our study highlights the suitability of a carefully planned multi-testing strategy for whole-genome screens for signatures of directional selection in natural populations.

## Supporting Information

Figure S1 **Overlap between SNPs outliers detected by the landscape genomics analysis for each of the environment characteristics.** Numbers within Euler diagram sectors represent number of SNPs, shown to be associated with an appropriate environment characteristic or combination of characteristics.
(TIF)

Figure S2 **SNPs outliers based on the landscape genomics analysis results, plotted along the genome.** Both all detected SNPs (orange) and unique SNPs (violet) for each environmental characteristic are shown as vertical lines plotted along the genome. For easier comparison of results, global results based on Designs 1-4 are shown as in Figure 4: vertical coloured shadings (green) show genomic regions, where two or three regions detected by kernel-smoothing-based designs overlap; chromosome numbers are given, chromosomes bearing regions exclusively containing design 4 “parasite outliers” are marked with red font colour, and “salinity outliers” - with blue.
(TIF)

File S1 **SNP allele frequencies per population.**
(XLSX)

Table S1 **Gene diversity and observed heterozygosity per SNP marker and per population.**
(XLSX)

Table S2 **Pairwise population differentiation as measured by** ***F*** **ST.**
(XLSX)

Table S3 **Analysis of molecular variance (AMOVA) results.**
(XLSX)

Table S4 **Significant genome region/outlier tests results for each of the 4631 SNPs.**
(XLSX)

Table S5 **Detailed list of all significant single test results with design 1, 2 or 3.**
(XLSX)

Table S6 **Significant genome region/outlier tests from each design that contributed to the six identified regions suggested to be affected by "parasite" or "salinity"- influenced selection.**
(XLSX)

Table S7 **List of the 2857 SNP loci with a significant BLAST match and GO annotation.**
(XLSX)

Table S8 **Significant BLAST match and GO annotation for the single outlier SNPs identified using Design4 and landscape genomics analysis.**
(XLSX)

Table S9 **Results of an enrichment test conducted for GO terms associated with the "Parasite" and “Salinity” subsets of SNP outliers identified using Design4.**
(XLSX)

Table S10 **Results of an enrichment test conducted for GO terms associated with the "Parasite" and “Salinity” subsets of SNP outliers identified using landscape genomics analysis.**
(XLSX)

Table S11 **Functional network analysis: comparison of GO terms associated with "Parasite" and "Salinity" sets of SNP outliers as identified by Design 4 and landscape genomics analysis.**
(XLSX)
