# Supplementary figures and images for: Footprints of Directional Selection in Wild Atlantic Salmon Populations: Evidence for Parasite-Driven Evolution?
Source: PLoS One. 2014 Mar 26;9(3):e91672. doi: 10.1371/journal.pone.0091672 (PMC3966780; doi:10.1371/journal.pone.0091672)

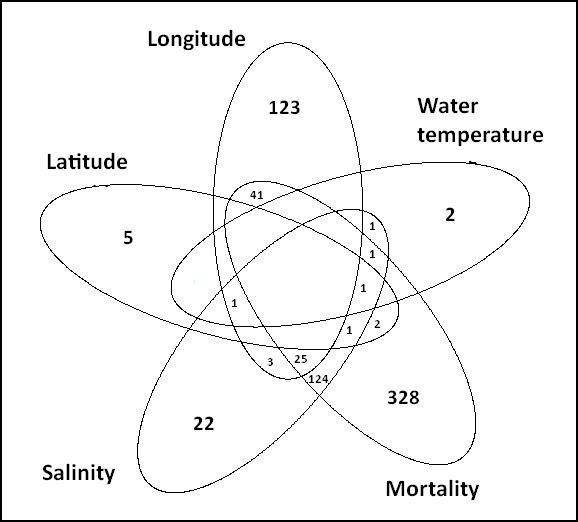

Supplement: Figure S1 — Overlap between SNPs outliers detected by the landscape genomics analysis for each of the environment characteristics. Numbers within Euler diagram sectors represent number of SNPs, shown to be associated with an appropriate environment characteristic or combination of characteristics. (TIF) [file pone.0091672.s001.tif]

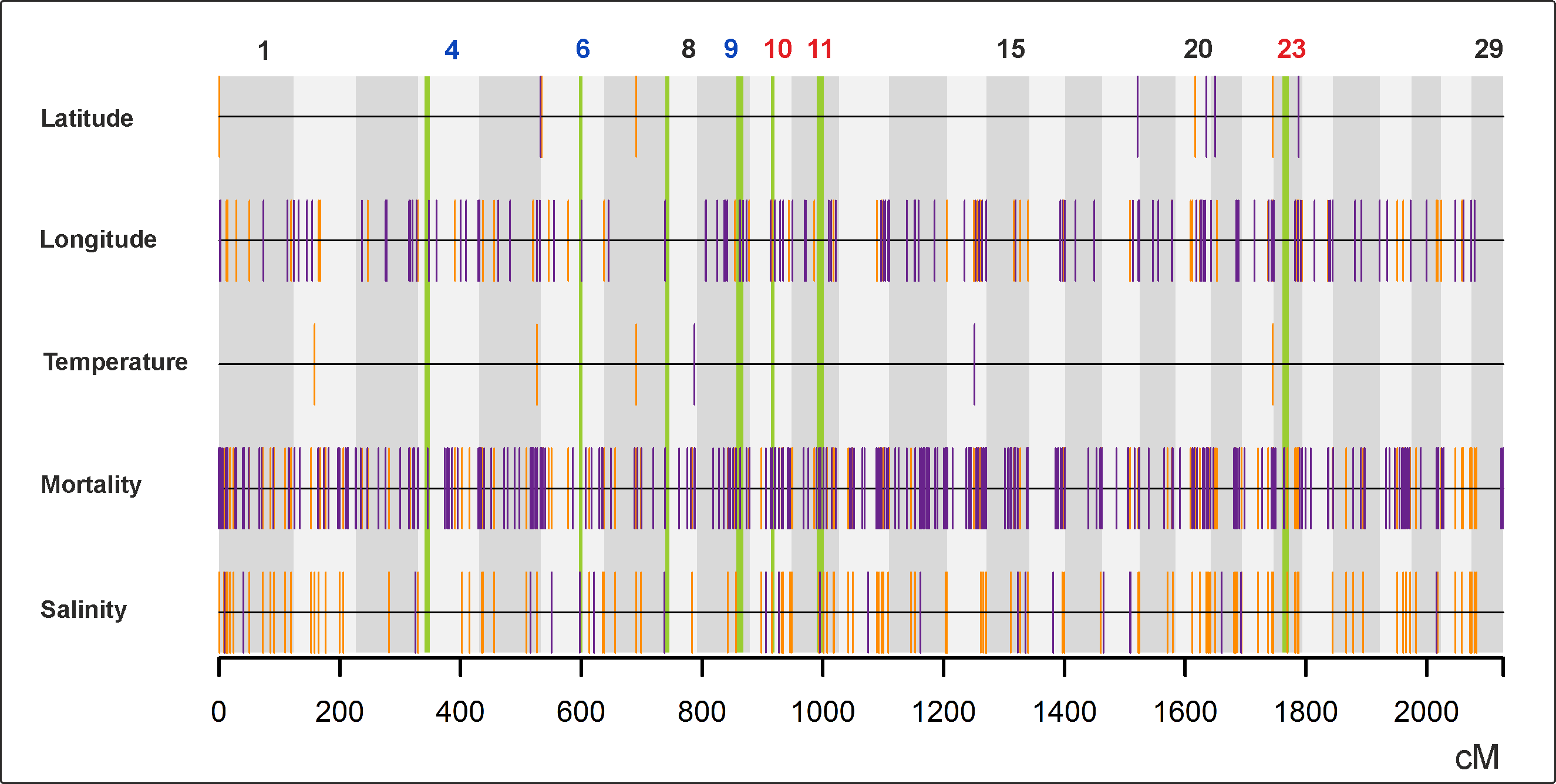

Supplement: Figure S2 — SNPs outliers based on the landscape genomics analysis results, plotted along the genome. Both all detected SNPs (orange) and unique SNPs (violet) for each environmental characteristic are shown as vertical lines plotted along the genome. For easier comparison of results, global results based on Designs 1-4 are shown as in Figure 4: vertical coloured shadings (green) show genomic regions, where two or three regions detected by kernel-smoothing-based designs overlap; chromosome numbers are given, chromosomes bearing regions exclusively containing design 4 “parasite outliers” are marked with red font colour, and “salinity outliers” - with blue. (TIF) [file pone.0091672.s002.tif]
